# Fracture Patterns in Fatal Free Falls: A Systematic Review of Intrinsic and Extrinsic Risk Factors and the Role of Postmortem CT

**DOI:** 10.3390/jcm14176305

**Published:** 2025-09-06

**Authors:** Filip Woliński, Kacper Kraśnik, Łukasz Bryliński, Jolanta Sado, Justyna Sagan, Katarzyna Brylińska, Grzegorz Teresiński, Tomasz Cywka, Robert Karpiński, Jacek Baj

**Affiliations:** 1 Department of Correct, Clinical and Imaging Anatomy, Medical University of Lublin, Jaczewskiego 4, 20-090 Lublin, Poland; rush22235@gmail.com (F.W.);; 2 Department of Forensic Medicine, Medical University of Lublin, Jaczewskiego 8B, 20-090 Lublin, Polandtomasz.cywka@umlub.pl (T.C.); 3 Department of Machine Design and Mechatronics, Faculty of 1 Mechanical Engineering, Lublin University of Technology, Nadbystrzycka 36, 20-618 Lublin, Poland; 4 Institute of Medical Sciences, The John Paul II Catholic University of Lublin, Konstantynów 1H Street, 20-708 Lublin, Poland

**Keywords:** fatal free fall, blunt force trauma, skeletal fractures, forensic pathology, postmortem computed tomography, autopsy, injury patterns, fall height, suicide, accident

## Abstract

**Background:** Free fatal falls (FFF) represent a distinct form of blunt force trauma (BFT) that is frequently encountered in forensic practice. Distinguishing FFF injuries from other forms of BFT, such as motor vehicle accidents (MVAs), can pose challenges. Despite its growing usage, the role of postmortem computed tomography (PMCT) in diagnosing FFF and its comparison with autopsy remains underexplored. **Purpose:** This review synthesizes fracture patterns in FFF, examining both extrinsic and intrinsic variables that influence skeletal injuries. It also compares PMCT and autopsy findings to establish a replicable database for forensic analysis. **Methods:** PubMed and Google Scholar were systematically searched by three independent reviewers. The inclusion criteria required studies to be published in English, report at least 10 cases, focus on fatal falls, and provide precise data on skeletal injuries. Studies lacking detailed descriptions, focusing on survivors, or involving non-free falls were excluded. Data extraction tables facilitated synthesis and analysis. **Key Findings:** FFF are characterized mainly by axial skeletal fractures, particularly of the chest, skull, and pelvis. A history of intoxication and psychiatric disorders often correlates with the manner of death. Fracture patterns vary by fall height, impact surface, and cause: accidental falls show greater chest and skull involvement, whereas suicidal falls present more pelvic and skull fractures. PMCT detects fractures more frequently than traditional autopsy. **Conclusions:** Distinct fracture patterns aid in differentiating suicidal from accidental FFF, shaped by extrinsic and intrinsic factors. Given its superior fracture detection capabilities, PMCT should be integrated into forensic protocols for FFF investigations.

## 1. Introduction

Fatal free falls (FFF) represent a specific category of blunt force trauma (BFT) commonly encountered in forensic practice. According to the World Health Organization (WHO), falls are the second leading cause of unintentional injury deaths worldwide, trailing only behind motor vehicle accidents (MVA). The causes of fatal falls can be classified into three categories: accidental, suicidal, and homicidal [1].

Accidental FFF predominantly affect men and often occur in workplace settings [2,3,4]. Suicidal FFF, typically associated with urban environments, victims are of low socioeconomic status, between 20 and 40 years old, usually involve falls from significant heights, and tend to happen late at night [5]. Homicidal FFF, while rare, are often poorly documented in the literature. Reports indicate that a disparity in strength between the perpetrator(s) and the victim is frequently a factor, although this is not always the case. Homicides may be staged to appear accidental. Differentiating between a fatal fall from standing height and homicidal blunt force trauma to the head is challenging, a difficulty compounded by the limited reliability of the “hat brim rule”. These complexities make it challenging to draw universal conclusions about homicidal FFF [6,7].

The pattern of injuries resulting from fatal free falls is nonspecific and influenced by numerous variables. Fatal falls are characterized by an uninterrupted trajectory, meaning the body does not strike any obstacles before impact. They are typically categorized into low falls (less than 3 m) and high falls (more than 3 m) [8]. However, the classification of FFF is broad, and the line between low and high falls is unclear. Fatal falls from standing height represent a distinct subcategory of low falls with notable forensic and legal relevance. Owing to their unique force transmission dynamics and injury patterns, they were excluded from the present review.

Injury patterns in fatal free falls are influenced by two categories of factors: (1) intrinsic variables, which are victim-related (e.g., age, sex, BMI, state of consciousness, psychiatric or medical history, intoxication, muscle tone), and (2) extrinsic variables, which are environment- or event-related (e.g., fall height, landing surface type, body position, weather, velocity, obstacles) [8,9,10,11,12,13,14].

While specific injuries may be characteristic of FFF, they are not pathognomonic; similar injury patterns can also occur in other forms of blunt force trauma. For instance, the effects of vertical deceleration in FFF closely resemble those of horizontal deceleration and acceleration seen in motor vehicle accidents. Forensic experts often face challenges when skeletal remains showing signs of BFT are discovered in remote areas, making it difficult to determine whether the victim was run over, beaten, or fell from a nearby structure, such as an old bridge or tree [1,15].

Most existing literature on skeletal injuries in FFF victims is derived from autopsy-based studies. In classical postmortem examinations, skeletal structures—such as the pelvis, spine, and bones of the upper and lower limbs—are either not fully exposed or are superficially examined. Consequently, most findings focus on broad areas commonly susceptible to BFT, while detailed analyses of specific skeletal injuries are often lacking. Although patterns of injury typical of suicides and accidents are generally described in the literature, the frequencies vary, and types of bone fractures frequently remain unidentified, resulting in gaps in our understanding.

Postmortem computed tomography (PMCT) is an increasingly popular method that supports or, in some instances, replaces traditional autopsies. PMCT enables a comprehensive examination of deep skeletal structures that may not be visible during a conventional autopsy. According to a systematic review by Jalalzadeh et al., PMCT is more effective at diagnosing skeletal injuries but less effective at evaluating organ and soft tissue damage compared to traditional autopsies [16,17,18].

Numerous reports do not distinguish between different types of falls (accidental, suicidal, homicidal) or account for crucial extrinsic (fall height, surface type, body position at impact) and intrinsic (age, sex, mental health status, intoxication) variables that significantly influence injury patterns. The lack of comprehensive, systematic reviews comparing PMCT and autopsy findings, while integrating contextual factors, represents a significant research gap. Addressing this gap is both timely and necessary, as it would facilitate the creation of a standardized, replicable injury profile database to strengthen forensic assessments, improve differentiation between accidental and suicidal falls, and support accurate reconstruction of equivocal death scenarios.

This review aims to analyze free fatal falls in an anatomical context, synthesize fracture frequencies to identify general fracture patterns and manner of death patterns and gather results regarding the main extrinsic and intrinsic variables that influence skeletal injuries, thereby creating a replicable database of all FFF autopsy and PMCT studies. Additionally, fracture frequencies obtained from PMCT and autopsy studies will be compared.

The clinical relevance of identifying specific fracture distributions in fatal free falls extends beyond forensic differentiation between accidental and suicidal events. Accurate mapping of skeletal injury patterns—particularly those revealed through PMCT—can inform emergency trauma protocols, optimize clinical suspicion of fall dynamics in unconscious patients, and support public health strategies for fall prevention. Moreover, enhanced understanding of injury mechanisms can contribute to the development of predictive models in trauma surgery and improve medico-legal assessments in cases of ambiguous etiology.

## 2. Methods for Literature Selection

**Figure 1:**
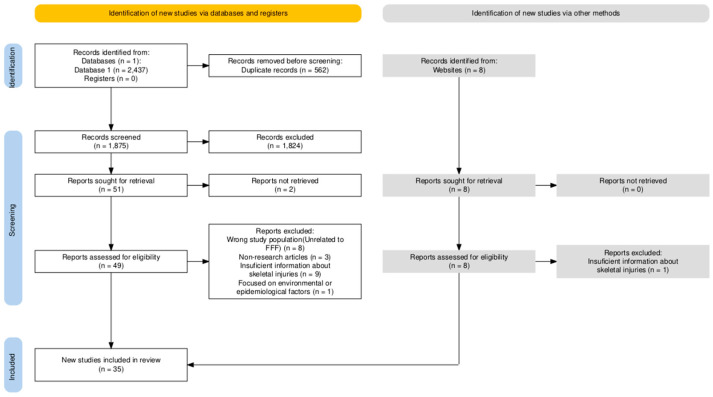
PRISMA 2020 flow diagram showing the process of study identification, screening, eligibility assessment, and inclusion in the systematic review.

This systematic review was conducted in accordance with the Preferred Reporting Items for Systematic Reviews and Meta-Analyses (PRISMA) 2020 Statement. The PRISMA 2020 checklist was completed to ensure that all relevant methodological and reporting standards were met. The process of study selection is presented in a PRISMA 2020 flow diagram (Figure 1). The review protocol was not prospectively registered in PROSPERO or any other database, as the research team determined that formal registration was not mandatory for the scope and nature of this forensic-focused review at the time of initiation.

The criteria for inclusion in this review were as follows:

- Studies published in English.
- Studies that comprise at least 10 cases were considered, as papers with fewer cases were deemed unlikely to provide reliable conclusions regarding skeletal injuries.
- Studies that focus exclusively on fatal falls, given that injuries from non-fatal falls differ in severity and nature.
- Clear descriptions of skeletal injuries, along with provided frequencies and patterns.
- Original studies featuring postmortem or PMCT findings.
- Full text available online (paywalled studies were included as long as the complete work was accessible).
- Publications in peer-reviewed journals.

The exclusion criteria were as follows:

- Studies published in languages other than English.
- Studies with fewer than 10 cases.
- Studies that describe injuries in survivors or hospitalized patients following falls.
- Injuries described in vague detail or reported only partially.
- Studies focusing on types of falls other than free falls—such as stair falls or interrupted falls. However, it is essential to note that many studies combine different types of falls; in our study, we decided that these can be included if they meet the inclusion criteria, provided that other types of falls account for a minority of cases.
- Studies about FFF that do not focus on the injuries.
- Conference abstracts, dissertations, organizational reports, and other forms of grey literature.

We searched using the PubMed database and Google Scholar, with data collected as of 20 December 2024. The search terms were “fatal free fall,” “fatal fall,” “fatal fall PMCT,” “fatal high fall,” and “fatal falls postmortem computed tomography,” combined using the Boolean operator “AND.” No filters were applied (e.g., publication date, study type). Grey literature sources, such as conference abstracts, dissertations, and organizational reports, were explicitly excluded to ensure a focus on peer-reviewed studies. When using Google Scholar, the first 1000 results of each keyword were inspected, as further research was unavailable (Figure 1).

All papers were screened and assessed independently by three reviewers. No automation tools were used to facilitate the search and selection process. Abstracts were initially evaluated for relevance, and studies deemed significant underwent a full-text review. Our team designed data extraction tables based on the existing literature and domain knowledge. These tables categorized skeletal injuries by major anatomical areas and specific bones. To ensure consistency, the tables were piloted independently on a subset of three studies by all reviewers. The piloting process identified discrepancies in terminology and categorizations, which were resolved through discussion. Based on these findings, the table layout and criteria definitions were refined to improve clarity and reliability. After piloting, the finalized data extraction tables were applied to all included studies. Relevant studies were added to a general database, and duplicates were removed. The inclusion/exclusion process was documented in a preformed tracking document.

Initial inclusion decisions were blinded, and discrepancies were resolved by vote, requiring agreement from at least two reviewers. All papers were subsequently added to a shared Mendeley folder. Where possible, intrinsic and extrinsic variables were collected from the reviewed studies, including sex, cause of the fall, fall height, type of landing surface, mental health conditions, and drug use. The victim’s body height, BMI, and primary occupation were not collected as their reporting is often lacking or condescending and goes beyond the scope of interest for this review. Heights reported in non-SI units (e.g., feet) were converted to meters using a conversion factor of 30.48 cm per foot.

We calculated the average percentages and values by synthesizing data from all included studies. To ensure accuracy, studies that did not report on a specific fracture type have been excluded from the corresponding calculations to avoid underestimating the true frequency. For example, only those studies that explicitly reported cervical spine fractures were included in the determination of the mean frequency of cervical spine fractures. Consequently, the resulting average reflects data from studies that specifically address this fracture type. Additionally, we include maximum and minimum values extracted from the works to provide context for the synthesized data. Despite the comprehensive search conducted independently by three reviewers, the possibility of missing relevant studies cannot be excluded. Moreover, excluding studies published in languages other than English and grey literature may have introduced selection bias, potentially limiting the diversity of findings. We also excluded studies with fewer than 10 cases, as small sample sizes were considered inadequate for meaningful comparison. Graphics illustrating the literature selection were made using an online tool [19].

The primary outcomes of interest were the types and frequencies of skeletal injuries observed in fatal falls and patterns of injuries typical for each cause. Effect measures included frequencies, percentages, and comparative proportions between autopsy and PMCT studies to identify differences in injury detection.

This review does not explicitly analyze injury patterns by fall height categories. While the severity of injuries generally increases with height, the relationship is complex and influenced by additional factors such as body position, landing surface, and energy transfer upon impact. Some studies have attempted to estimate fall height based on injury severity using mathematical models, but this remains a separate field of forensic analysis, explored in other dedicated reviews and studies [20,21].

In line with established recommendations for conducting systematic reviews, a formal appraisal of the methodological quality of the included studies was undertaken. Three reviewers independently assessed each study according to criteria derived from the Newcastle–Ottawa Scale (NOS). The appraisal addressed study design, clarity of inclusion criteria, completeness of outcome reporting, and the potential risk of bias.

The NOS assesses three domains: (1) Selection of study groups (up to 4 points), (2) Comparability of groups (up to 2 points), and (3) Outcome/exposure assessment (up to 3 points). Consequently, each study can attain a total score ranging from 0 to 9, with higher scores denoting a lower risk of bias. In this review, studies were classified into three categories based on their risk of bias: low risk (7–9 points), moderate risk (5–6 points), and high risk (0–4 points). When information was unclear or omitted, the item was scored as 0. The quality assessment was conducted independently by two reviewers, with any disagreements resolved through discussion until consensus was achieved.

Studies were awarded points for including only fatalities, utilizing autopsy or PMCT for confirmation, and possessing a relatively large sample size of at least 200 cases. Reports lacking clarity regarding consecutive inclusion or jurisdiction-wide coverage received a score of zero. The assessment of comparability relied on whether studies stratified cases based on pertinent variables, such as sex and fall height (1 point), and either fall intent or surface type (an additional 1 point), with a maximum achievable score of 2 points. The evaluation of outcomes was based on the methods used to measure and analyze fracture patterns, with points awarded for the use of standardized assessment techniques (autopsy/PMCT) and for the application of appropriate statistical analyses. The use of blinded or independent assessments was seldom documented and consequently scored zero.

## 3. Intrinsic Variables

Table 1 summarizes the intrinsic (victim-related) factors, including demographics, psychiatric disorders, and toxicology findings, which may influence injury patterns. Sample sizes ranged from 10 to 1002 cases, with a mean of 216.97 and a median of 143. Collectively, the papers describe 7594 individuals across various circumstances of death and patterns of injury. Male-dominated populations were consistently reported, with men comprising an average of 71.96% of cases (median: 72.38%). Similarly, the absolute number of autopsies performed for fatal falls reflected this male predominance, with men averaging 171.27 (median 106) and women averaging 69.88 (median 36) in the related studies.

Fatal falls were categorized as either accidents or suicides, with both categories receiving similar research attention about the total cases studied. Accidents accounted for an average of 46% of cases (median 53.03%), while suicides represented 46% (median 45%). However, in absolute terms, accidents significantly outnumbered suicides, with a mean of 146.45 cases for accidents compared to 75.1 for suicides—a nearly 2:1 ratio. This discrepancy suggests that while studies examine both categories proportionally, accidents are more frequently represented in total case numbers. Homicidal fatal falls were uncommon in the studies reviewed, with cases ranging from 1 to 9. Most studies did not offer detailed information on the victims’ sex in these instances, which limited further conclusions. On average, the cause of the fall was undetermined in 3.78% of cases, corresponding to an average of 7.45 instances per study. Notably, only 26.47% of the papers reported such cases; in the remaining studies, authorities identified a definitive cause of the fall for all victims.

Analysis of the gender data reveals that, of those who used fatal falls as a method of suicide, men constituted the majority, accounting for an average of 53.74% and a median of 56% of cases. In contrast, women accounted for an average of 31.39% and a median of 35.04%. This trend is even more pronounced in accidental deaths, where males represented an average of 56.8% and a median of 73.05%, while women made up an average of only 10.94% and a median of 10%. However, these conclusions should be interpreted with caution, as 18 papers did not provide sex-specific data for individual causes of death, which introduces potential bias in the analysis.

Reporting on toxicology varied across studies. Note that many included articles did not include information on whether toxicology screening was performed in every FFF case included, which may introduce bias. The results were simplified into three categories. Psychoactive substances (at least one of the categories such as alcohol, drugs, and other illegal substances) were considered in 10/35 studies (28.57%), with an average prevalence of 24.39% (ranging from 1.41% to 81.12%). Among these, prescribed medications—primarily psychiatric drugs—were the most common, showing an average prevalence of 20.84% (ranging from 1.41% to 73.52%). Alcohol consumption was reported in an average of 8.5% of cases, reaching a maximum of 24.39%. Other illicit substances, such as morphine, amphetamines, and cocaine, were noted in an average of 4.02% of cases, with a median of 1.41%. In four articles, we gathered information about the prevalence of intoxication among manner-of-death groups. Generally, suicide victims were more frequently under the influence of psychoactive substances, with an average of 34.08% for alcohol across all suicides, 6% for drugs, and 12.45% for other illicit substances, in comparison to an average of 10% for alcohol and 5.65% for illegal substances in accidents. The four studies mentioned had either a significant dominance of accidents or suicides, which may introduce bias into the averages.

Psychiatric disorders were reported in 11/35 articles (31.43%), with an average prevalence of 17.87%. The most commonly reported conditions were substance use disorder or a history of overdose and depressive disorder, each mentioned in four studies. Schizophrenia was reported with a prevalence ranging from 0.83% to 1.47%, depressive disorder varied from 2.27% to 24.14% (mean 9.97%), and substance use disorder or history of overdose ranged from 0.78% to 10% (mean 5.57%). It should be recognized that the victim’s medical history is not always available. Moreover, many cases of psychiatric disorders are not diagnosed or treated. Thus, the data presented above must be interpreted with caution.

## 4. Extrinsic Variables

Table 2 summarizes the extrinsic (event-related) factors, such as fall height, landing surface type, and use of PMCT, that may modify injury distribution. Data on fall heights were unavailable in 4/35 studies (11.43%). Notably, each study employed its classification system for fall heights (e.g., <6 m and >6 m), making the consistent identification and comparison of low (<3 m) and high (>3 m) falls challenging. Nonetheless, our analysis is presented below.

Many studies included a mixed group of high (>3 m) and low (<3 m) free falls. Low falls were absent in 37.14% (13/35) of the papers. Among the studies that included low falls, the lowest reported height ranged from 0 to 0.6 m, with only one study (2.86%) focusing exclusively on falls of this height. Conversely, 37.14% (13/35) of the papers examined only high falls. The highest reported fall in the sample occurred from a height of 200 m, establishing a range of 0–200 m for fatal falls across the included studies.

In terms of height categories, 62.86% (22/35) of papers included falls within the 10–20 m range, 51.43% (18/35) included falls in the 20–50 m range, and 48.57% (17/35) reported falls in the 50–100 m range (although most cases were under 60 m). Falls greater than 100 m were reported in 14.29% (5/35) of studies. When considering only studies that explicitly distinguished between falls of greater than 3 m and those of less than 3 m, high falls predominated in 51.43% of papers (18/35). In contrast, low falls accounted for just 5.71% (2/35). The remaining 42.86% (15/35) of studies used height ranges that could not be categorized as exclusively high or low falls.

Regarding fall surfaces, hard or non-deformable surfaces were the most commonly reported, with a mean prevalence of 69.74%. Soft or deformable surfaces appeared in 0.07% of cases, while water surfaces had an average prevalence of 26.67%. Other surface types were mentioned less frequently, with a mean of 1.75%.

## 5. General Fracture Frequencies and Their Anatomical Distribution

### 5.1. Skull Fractures

Across all studies, skull fractures were reported in 2785 out of 7594 cases (36.67%), with a mean prevalence of 55.44% and a median of 59.05%. However, not all studies systematically reported skull fractures.

- The cranial vault and skull base were fractured with similar frequency, with an average prevalence of 29.36% for vault fractures and 25.06% for skull base fractures.
- When studies combined vault and base fractures, the reported prevalence was 29.86%. Facial skeleton fractures were observed in 0% to 32.5% of cases, with an average prevalence of 12.79%. Four studies mentioned the occipital “ring fracture,” often associated with deceleration injuries, nine cases were reported. Mandible fractures were reported in two studies, acounting for 9.4% and 15% of all fractures, respectively.

### 5.2. Spinal Column Fractures

The spinal column was reported in 80% (28/35) of the included studies, accounting for 778 victims (10.25%) out of a total of 7594 cases.

The spinal column was fractured with an average prevalence of 24.79% throughout the studies and a median of 16.45%.

- The cervical spine was fractured in an average of 14% of cases, which had referred spinal fractures (median: 12.2%, range: 1.1–50%).
- Upper cervical fractures were more common than lower cervical fractures (59.89% vs. 40.11%). Among upper cervical fractures, C1 was fractured in 21.1% of cases, while C2 fractures accounted for 62.39%, and 16.5% of victims experienced C0/C1 dislocation. Dens fractures were nearly twice as common as arch fractures. Thoracic spine fractures occurred in an average of 16.46% of cases in related studies (median: 10%, range: 1.3–52.27%). Lumbar spine injuries were reported in an average of 6.8% of cases (median: 4.1%), making it the least commonly injured spinal region, with only 145 cases reported. Thoracolumbar fractures were reported with rates of 31.2%, 42.1%, and 67.9%. The average prevalence was 47.07%, affecting 253 victims. Only one study separately examined the sacrum and coccyx, with fractures reported in 9% (28/307) of cases.

### 5.3. Pelvic Fractures

Pelvic fractures were described in 23 out of 35 studies (65.71%), involving 1239 victims (16.32%) of the total 7594 cases. Different studies described the locations of pelvic fractures with varying degrees of precision.

- Pelvic fractures were observed in an average of 29.56% of cases related to this injury (median: 19.78%), with reported frequencies ranging from 1.1% to 100%.
- The pubic symphysis was disrupted in 4.8% to 36% of cases across the two studies that examined it. Sacroiliac joint damage was noted in three studies with an average prevalence of 22.07%.
- One study reported pubic bone fractures in 13.97% of cases, while another found fractures in 22% of the ilio-pubic rami and 16% of the ischio-pubic rami.

### 5.4. Upper and Lower Limbs Fractures

Upper limb fractures were noted in 70.83% (17/24) of studies, comprising 427 cases (5.62%).

- Upper limb fractures occurred in an average of 36.72% of cases throughout related studies (median, 20.7%; range, 5–100%).
- Clavicle fractures showed a mean prevalence of 7.69%, scapula fractures 2.74%, humerus fractures 11.19%, forearm fractures (radius and ulna) 10.52%, and hand fractures 2.52%.

Lower limb fractures were documented in 66,67% (16/24) of studies, totaling 563 cases (7.41%).

- Lower limb fractures were observed with an average prevalence of 39.18% in studies related to this topic (median: 29.07%, range: 8–100%).
- Femur fractures showed a mean prevalence of 13.47%, shin fractures a prevalence of 10.01%, and foot fractures a prevalence of 5.23%. Seven cases of calcaneal rupture were observed.

Two studies also reported joint injuries, with hip joint damage occurring in 1% to 3.42% of cases, knee joint injuries in 1.37%, elbow joint injuries in 5.48%, ankle joint injuries in 2.06%, sternoclavicular joint injuries in 3%, and shoulder joint injuries in 2%.

### 5.5. Chest Fractures

Thoracic injuries were reported in 77.14% (27/35) of the included studies, accounting for 38.6% (2931/7594) of all cases.

- Rib fractures emerged as the most frequently reported skeletal injury in fatal falls, occurring in an average of 65.67% of cases (median 71%).
- Fractures of the right and left ribs were noted at comparable rates (14.34% and 14.63%, respectively).
- Bilateral rib fractures had a mean prevalence of 33.18% (median: 37.88%), while unilateral fractures accounted for an average of 26.76% of cases (median: 25.61%).
- Sternal fractures were documented with an average prevalence of 19.49% (median, 17.3%; range, 3.3–46%).

### 5.6. Patterns of Injury

Fatal free falls exhibit distinctive injury patterns influenced by both intrinsic and extrinsic variables. Among our sample, we observed overlapping proposed patterns related to the impact position (feet/buttocks, head, side/whole body) and the cause of the fall (accident/homicide/suicide). As in other sections of this review, we included studies that reported injury frequencies. Refer to the discussion chapter for the combined conclusions from studies that statistically analyzed the injury patterns without offering specific frequencies.

**Figure 2:**
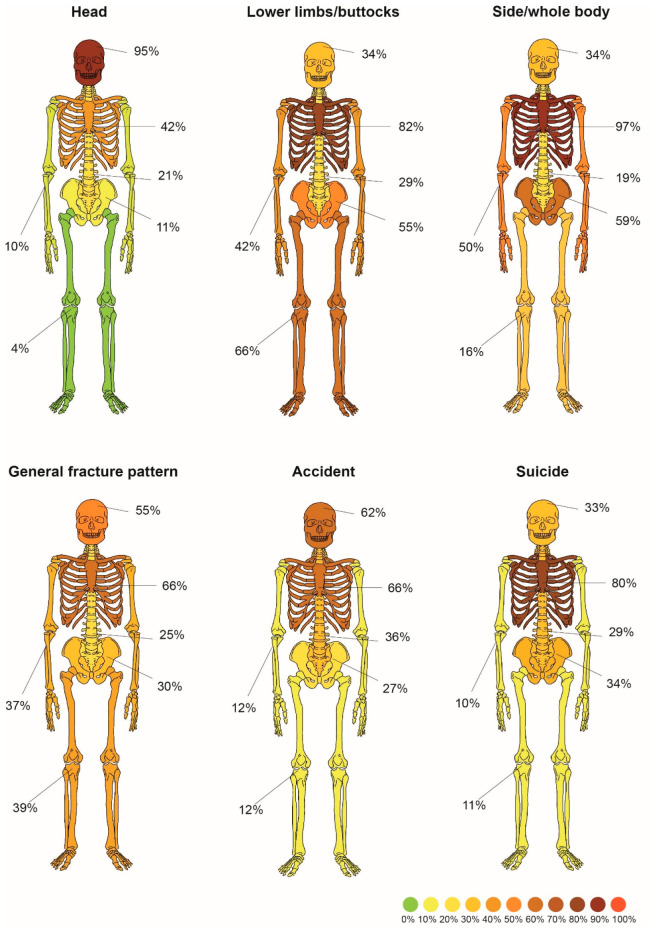
Synthesized fracture patterns from the literature—general fracture pattern, impact positions, accidents, and suicides.

Figure 2 presents the synthesized injury patterns derived from the reviewed studies, categorized by impact position (head, feet/buttocks, lateral/full body) and fall type (accidental, suicidal), based on comparative fracture frequency data.

By collecting data from the literature, several fracture patterns caused by FFF were isolated. Highlighted fracture patterns due to causes of FFF, such as accidents and suicide (homicide was passed over due to a small group of cases). Additionally, a fracture pattern for the impact position (head, lower limb/buttocks, side/whole body) and one general pattern (summarizing all described cases without differentiation by cause of FFF and impact position) were identified.

## 6. PMCT Detected Fracture Frequencies

Postmortem computed tomography was employed in 5/35 studies, accounting for 14.29% of all included papers. The synthesized fracture frequencies from these studies were as follows:

- Skull 83%
- Chest 95%
- Spine 79%
- Pelvis 83%
- Upper limbs 78%
- Lower limbs 69%

Refer to the discussion for a detailed breakdown and a comparison to autopsy data.

### Risk of Bias Assessment

The risk of bias assessment according to the NOS is presented in Table 3. 

Overall, the majority of studies were characterized by moderate methodological quality. The most frequently identified limitations comprised small sample sizes, heterogeneity in fracture classification systems, and incomplete reporting of potential confounders (e.g., psychiatric history, results of toxicological testing). Such structured quality assessment facilitates a more nuanced interpretation of the evidence’s strength, underscoring that the most robust conclusions stem from studies of high methodological quality, whereas findings from studies of lower quality warrant cautious interpretation.

## 7. Discussion

This review provides an overview of skeletal injury patterns in fatal free falls, emphasizing factors such as the cause of death, demographic trends, and fracture distributions. Please note that this review is primarily based on research articles detailing injuries, so the information regarding other variables may be incomplete.

### 7.1. Population

The current literature indicates that FFF demographics are predominantly male. This is reflected in our synthesized results, where males outnumber females by a ratio of 3 to 2 [50].

FFF are predominantly accidental, with accidental cases significantly outnumbering suicidal ones. Only one study reported more female than male suicides [29]. In the context of suicide, men also tend to choose more violent and high-lethality methods, such as FFF, at a higher rate than women. Moreover, some studies suggest that suicidal males tend to select greater heights for jumps than females [24]. However, other studies report similar male-to-female ratios across all height categories [34]. This effect needs further study. A separate subject is the geographical distribution of FFF, as in many countries, suicide is prohibited by religion. Thus, a lower suicide/accident ratio makes it difficult to draw universal conclusions [42,51]. Studies included in this review consistently show a predominance of male fatalities in FFF cases, supporting the notion that both accidental and suicidal falls disproportionately affect men [5,28,34,35,50,52,53,55].

It has to be noted that according to a study focused on intrinsic variables in FFF, sex does not affect the fracture pattern [50]. However, another study noted a higher frequency of pelvic fractures in females due to anatomical distinctness from males (wider build, greater flexibility of ligaments); for unclear reasons, this effect also involved cervical vertebrae; rib fractures were more common in males, probably due to higher BMIs [41]. Further research is needed to elaborate on those findings.

### 7.2. Toxicology

According to studies, intoxication is prevalent among FFF victims. As many as 70% of FFF suicides and 36% of accidents may test positive in toxicological analyses. Psychoactive medications are frequent in suicides, while alcohol in accidents. Therefore, the results of toxicological analyses may indicate the manner of death, particularly in cases without a clear social or psychiatric history. Notably, some studies contradict this pattern, indicating that women may be more likely than men to commit suicide while under the influence of alcohol [56,57,58]. This highlights that detecting intoxication could be an indicator but not a definitive assertion.

Collected papers seem to confirm that psychoactive substances are more frequently present in suicides. Where only four studies described the exact number of suicides committed under the influence of alcohol, analogously, three studies were referring to accidents [26,30,32,45]. Interestingly, analysis of these studies showed that alcohol intoxication appears to be more prevalent in suicides than in accidents, which contradicts some findings from previous studies. Several factors may explain this discrepancy. First, only four studies in the dataset directly compared the toxicological profiles of suicides and accidents. Second, we excluded studies where toxicology is the primary focus. Third, the relationship between alcohol intoxication and fall-related fatalities may be more complex—as indicated by the articles contained in this review—and much depends on the proportion of accidental versus suicidal falls, geographic and cultural factors (e.g., rural versus urban settings), and religious or societal norms affecting substance use. For instance, a study from a predominantly Muslim country like Egypt or Tunisia is likely to report few to no cases of alcohol intoxication, as its consumption is not culturally or religiously accepted by locals [51,54].

One study linked intoxication with more upper limb fractures [43]. Conversely, another study found no effect of intoxication on injury patterns in high falls, while it identified an increased risk of occipital fractures in ground-level falls [36]. According to Papadopoulos et al., alcohol consumption is one of the most common pre-injury factors [37]. Most works report numbers but do not analyze the effects and correlations between injury and intoxication. This is a possible area for future studies.

### 7.3. Psychiatric Disorders

A history of severe psychiatric disorders is predominantly associated with suicidal FFF, with little to no presence in accidental cases [59]. However, distinguishing between suicidal and accidental falls can be challenging, particularly in cases involving altered mental states [52]. Conditions like psychosis or substance-induced delirium can cause individuals to develop irrational beliefs may result in high-altitude jumps. Such cases blur the line between accident and suicide. For this review, we classified such cases as suicidal FFF. Future research should investigate whether this subgroup has unique characteristics that set it apart from typical suicides and accidents. A study by Rowbotham et al. points out that psychiatric disorder in medical history does not affect the fracture pattern, yet further research is needed to facilitate this conclusion [47].

As expected, psychiatric disorders had a higher-than-normal prevalence in our sample, accounting for over half of the suicides in some articles [30]. Among documented psychiatric conditions, depressive disorder was the most frequently reported. Falling has historically been a common method of suicide, particularly in urban environments. Based on the reviewed literature, cases involving individuals with depressive disorder most often occurred in city settings [55]. The causes for choosing this method of suicide in other mentally ill patients are not clear.

Overall, reviewed articles suggest that in ambiguous cases, a documented history of psychiatric disorders increases the likelihood of a suicidal manner of death. However, psychiatric history should be interpreted alongside other forensic evidence rather than used as a sole determinant of intent [22,29,41,52].

### 7.4. Heights and Damage Profile

A primary factor influencing fall-related injuries is the body’s kinetic energy at the moment of impact. This energy comes from gravitational potential energy (PE), represented by the following equation:

PE = m × g × h

In this formula, many variables, like air resistance, were omitted for simplicity, which served only for comparison between cases and as a variable that considers both mass and fall height. Moreover, the limitation in calculations caused by air resistance is significant above a certain fall height, which was not exceeded by any of the considered cases.

Where m is the body’s mass, g is the acceleration due to gravity (9.81 m/s^2^), and h is the height of the fall. During free fall, this PE converts into kinetic energy, ultimately determining the impact force. Based on this relationship, fall height is expected to influence the severity, number, and distribution of fractures [33].

Most studies agree that an increase in fall height correlates with a rise in both the severity and frequency of fractures [34,37,38,39,40,41,49,54]. However, certain studies have not confirmed this association, a discrepancy likely attributable to the heterogeneity of study populations [47]. One study indicates that the number of affected body areas remains unchanged with increasing fall height [54]. This phenomenon may be explained by the tendency for injuries to cluster around the primary impact site. For instance, during feet-first landings, fractures primarily occur in the lower limbs and axial skeleton, which encompasses two or three body areas. In contrast, during head-first impacts, injuries are concentrated in the skull and other parts of the axial skeleton. Additionally, the high altitude of the fall was connected numerous times to the suicidal intentions [29,32,37,47,52].

Regardless of the type of impact, the kinetic energy of a fall is primarily transferred through the axial skeleton, making it more susceptible to fractures than the appendicular skeleton. This observation is helpful because it distinguishes pure bludgeoning—where one would expect blunt injuries on various body surfaces without significant trunk fractures—from free fall fatalities, where fractures of components of the axial skeleton (pelvis, chest, and skull) often co-occur.

Additionally, fractures of the axial skeleton increase with the height of the fall in both low and high-free falls [29,40,43,47].

#### 7.4.1. Chest

Rib fractures are highly likely in fatal free falls, particularly from greater heights. The chest is frequently reported as the most commonly fractured skeletal region in adult fatal free falls. Although rare, instances of FFF without thoracic involvement should prompt consideration of alternative mechanisms of blunt force trauma. Fractures of the ribs and sternum can also contribute to death, as they have the potential to damage internal organs such as threatening the lung, heart and other nearby organs [22,23,25,27,30,38,40,41,46,47,49].

The relationship between rib fractures and fall height remains a topic of controversy. Most studies suggest that the incidence and number of chest fractures—especially bilateral fractures—increase with fall height [24,29,38,41,43,47,49,50,53,54]. While others do not support this correlation [40,46]. This discrepancy may again be due to population heterogeneity. Moreover, some studies showed a higher frequency of rib fractures from lower heights. This effect may be explained by a higher initial survival rate from lower falls and, thus, more frequent resuscitation attempts, which lead to rib fractures [46]. Rib fractures can indicate the position of impact. Likewise, sternal fractures imply anteroposterior compression of the rib cage [60]. Multiple rib fractures across different anatomical planes have even been described as a “hallmark of bridge jumpers” [61]. In pediatric victims, the higher center of mass predisposes to skull fractures, which occur more frequently than rib fractures [62].

#### 7.4.2. Skull

Studies have identified three distinct distributions of skull fractures based on the height of the fall. In some instances, cranial injuries occur more often in falls from heights less than 10–15 m, while relatively few skull fractures are seen in the 10–20 m range, then reappear in falls from heights above 20 m, presenting a bimodal distribution. This seems to be biomechanically determined by the interplay between initial posture and body rotation during free fall. Pascoletti et al. [63] demonstrated through multibody modeling that the final impact configuration is governed primarily by the starting position and momentum, with limited potential for reorientation once in free flight. Experimental studies by Jones and Cory [14] further showed that small differences in fall initiation (e.g., hanging versus being pushed) lead to markedly different angular velocities and rotational trajectories, with head-first impacts more likely in short falls and multiple body rotations occurring in longer falls. These findings provide a plausible mechanistic explanation for our results: at low heights, limited time to rotate favors direct head-first landings and high skull fracture rates; at intermediate heights, the body often stabilizes into feet-first or side-first impacts, reducing cranial injury; and at very high falls, even feet-first impacts transmit sufficient energy or allow additional rotations to reintroduce cranial fractures. Thus, the bimodal fracture pattern reflects not only fall height but also the kinematic constraints imposed by human body dynamics during descent [24,33,40].

Nevertheless, this distribution pattern is not consistently supported across all studies. Many indicate a more linear relationship, where skull fractures consistently rise with increased fall height [43,50]. We hypothesize that this discrepancy may be explained by the accident/suicide ratio. In articles primarily focusing on suicides, we observe a more linear distribution due to the variability of impact positions. Suicides happen from various heights, with a plethora of landing positions; thus, in statistical analysis of skull fractures, we observe the working of the primary determinant of injuries—kinetic energy. At the same time, accidents tend to show a bimodal distribution, as accident victims usually fall from lower heights and rotate mid-air in more predictable patterns (falling back or forward during a slip). Further research comparing these groups is necessary to validate this conclusion. Not all articles found a correlation between skull fractures and the height of the fall, and in some cases, this relationship was inversely proportional [31,38,53,54].

In general, fractures of the skull vault and base occur similarly, with differences between the two rarely exceeding 10% in individual studies. Some research suggests that skull base fractures typically result from falls from heights greater than 3 m. On the other hand, different studies suggest that skull vault fractures are indirectly related to fall height, with the highest proportion occurring in falls from low or ground level. This may be explained by differences in body positioning at the moment of impact. Moreover, skull vault fractures usually happen near the impact zone. In contrast, skull base and cervical spine injuries tend to develop in areas remote from the point of impact, likely due to force transmission through the axial skeleton. Given this, skull base fractures may serve as a forensic clue, suggesting a fatal free fall rather than a ground-level fall. However, this effect is not supported by all the data [31,36,38,39,44,51]. Skull fractures can also help to differentiate fatal free fall from pedestrian impacts, as they are significantly more common among FFF [15].

Facial skeleton fractures in falls were studied in detail only from the non-forensic perspective [64]. Reviewed papers on facial skeleton fractures reveal inconsistent findings. Some studies suggest an inverse relationship between the height of falls and facial fractures, proposing that victims may instinctively turn their faces away from the point of impact. Conversely, other studies indicate a positive correlation between facial fractures and fall height. Furthermore, a connection between facial fractures and a victim’s BMI has been noted, suggesting a more complex interaction between extrinsic and intrinsic factors [38,40,44,49].

In some articles, the skull is more frequently fractured than the chest. This might relate to the ratio of various impact positions. However, since this parameter is not usually accessible for examination, it is difficult to determine. Further studies comparing different groups are necessary to explore this effect [29,31,42,51,53].

In a deceleration injury, such as a fatal free fall, the point of contact on the skull bends inward (inbending) upon impact, while the surrounding areas undergo outward bending (outbending). This deformation creates distinct zones of compressive and tensile stress. Specifically, inbending generates compressive forces on the outer skull surface and tensile stress on the inner surface, whereas outbending produces tensile forces on the skull’s outer surface.

Linear skull fractures typically originate in areas of maximum tensile stress caused by outbending, often at a distance from the impact site. These fractures tend to propagate toward the impact zone, and in some cases, extend in the opposite direction. The energy of the fall plays a crucial role in determining the fracture pattern: low to moderate energy may result in a single linear fracture. At the same time, higher-energy impacts commonly produce multiple fracture lines or stellate (radiating) patterns.

In high-energy impacts, the inbending at the point of contact can cause radial fractures from the impact center. Additionally, curvilinear or circular fracture lines may form at the junction between inbended and non-inbended bone.

When the occipital region or lateral aspects of the skull strike a surface during a fall, the resulting basilar fractures are typically caused by tearing-apart forces generated by the skull’s rapid deceleration. Depending on the impact location, these fractures may extend anteroposteriorly (from front to back) or transversely across the base of the skull. Such patterns are consistent with both experimental data and clinical observations of fatal head injuries [65].

In a study by Rowbotham et al. focused on low FFF, three fracture morphologies were distinguished, with a positive correlation to the height fallen. They were a linear skull fracture, a linear base fracture, and diastasis. The same study also disproved the notion that only high-energy events can cause fractures above the brim-of-hat line.

Occipital ring fractures are intriguing. These fractures result from the transmission of force through the axial skeleton and are often considered characteristic of fatal free falls. However, we found only nine documented cases after reviewing the included studies. This low incidence raises questions about the diagnostic reliability of occipital ring fractures as an indicator of fatal free falls. While they appear highly specific to certain conditions, their rarity suggests that they are not a reliable marker for determining the cause of death. Therefore, while occipital ring fractures should not be expected in every fatal free fall autopsy, they remain a relevant finding when they are present [22,30,31,32].

#### 7.4.3. Spine

Although the spinal column due to impact position experiences both direct and indirect transferred by the axial skeleton mechanical forces in most fall-related impacts, it ranks third—or occasionally even fourth or fifth—in terms of fracture frequency.

While some studies indicate a positive correlation between fall height and spinal injuries [24,29,43,44], others do not find a significant association [53,54]. Much depends on the impact position and other variables present [66]. Additionally, research is divided on which spinal regions are most affected, with cervical, thoracic, and lumbar vertebrae most commonly reported [31,40,47,49].

FFF have been linked to cervical spine injuries, including C0/C1 dislocation [39]. It was also positively correlated with the height of the fall, occurring eight times as prominently at altitudes greater than 3 m. This is best explained by high PE and an impact-specific occipital impact position, along with a flexed neck. This scenario produces a force vector perpendicular to the long axis of the spine, allowing for such an occurrence. Cervical vertebrae and clavicle fractures have been found to correlate with hyoid bone fractures, suggesting a potential pattern of force transmission in cases involving high-impact falls. What is more, lower cervical fractures correlate with the height of the fall [39,45]. Not all articles support the aforementioned conclusion; for instance, a study by Gupta et al. [24], cervical fractures dominated in the lower height group, while thoracic in high falls, which authors attribute to direct forces breaking cervical vertebrae and additional indirect forces engaged in thoracic segment injuries.

Spinal injury patterns vary according to the victim’s landing position. In the case of feet or buttocks impact, it typically results in compression; with side impact, it leads to angulation, while head-first impacts involve a combination of both [22].

Most studies included in our review rely on autopsy data, which may contribute to these discrepancies. Traditional postmortem examinations primarily focus on major skeletal injuries, potentially underreporting less obvious vertebral fractures. Given these methodological limitations, the true prevalence and anatomical distribution of spinal fractures in fatal falls remain uncertain. Advanced imaging techniques, such as postmortem CT scans, may offer a more comprehensive assessment and help clarify the relationship between fall height and spinal injuries.

#### 7.4.4. Pelvis

Pelvic fractures are particularly dangerous because they can lead to severe hemorrhage. They most commonly occur during feet-first, buttocks-first, or whole-body impacts, which result from direct horizontal deceleration or indirect vertical force transmission—mainly through the femur. As a result, injuries tend to concentrate around the main joints—sacroiliac joints and pubic symphysis. As a result, the pelvic injuries involve the sacroiliac joints and the pubic symphysis, although they differ structurally, being partly fibrous and cartilaginous, respectively [22,38,40].

Age and height increase risk of pelvic fractures. As with other injury patterns discussed in this review, the relationship between fall height and pelvic fractures remains controversial. Some studies report an increase in pelvic fractures with greater fall height, while others find no significant correlation. Advanced age is strongly associated with a higher risk, as weakened bone structure makes older individuals more susceptible to pelvic fractures. The relation between sex and pelvic fractures has been discussed in other paragraphs. Notably, in cases of suicide jumps, the probability of sustaining a pelvic fracture increases with every additional meter of fall height. Therefore, the pelvic ring may give us a clue about the manner of death and the body’s impact position [22,37,38,40,43,49].

Pelvic fractures can serve as valuable indicators of the direction and magnitude of force involved in trauma, potentially offering insights into the victim’s landing position in FFF. In orthopedic surgery, the Young and Burgess classification system distinguishes three primary mechanisms of pelvic injury: anterior-posterior compression (APC), lateral compression (LC), and vertical shear (VS) [67].

In APC injuries, the trauma typically begins with failure of the anterior pelvic ligaments, followed by damage to deeper structures. The symphyseal ligaments are initially disrupted by injury to the pelvic floor ligaments—the sacrospinous and sacrotuberous ligaments. The posterior sacroiliac complex is the last to be injured [68].

LC injuries, in contrast, are more frequently associated with pelvic fractures. They typically present with coronal-plane fractures of the pubic rami and may also involve sacral ala or iliac wing fractures. These fractures result from lateral impact forces that compress the pelvis from one side [69].

A cephalad-directed axial load causes VS injuries to one hemipelvis. This mechanism is commonly observed in falls from significant heights or motorcycle accidents, where asymmetrical leg loading occurs. The result is a cranial displacement of the iliac wing relative to the sacrum, accompanied by disruption of the symphyseal ligaments, pelvic floor structures, and the posterior sacroiliac complex [70].

The pelvis is not routinely explored during standard autopsies, leaving a gap in our understanding of how specific injury mechanisms correlate with impact positions in fatal falls. This area could benefit from applying advanced imaging techniques like postmortem computed tomography. Future investigations using PMCT may provide deeper insights into the injury mechanisms associated with different types of falls and could improve the accuracy of forensic reconstructions.

#### 7.4.5. Upper and Lower Limbs

Data on limb fractures are inconsistent [31,53]. However, this is not accounted for in other articles that state that limb fractures correlate with the height of the fall, the overall chance for four limb fractures increases [24,38,44,71]. That might be true for some cases, but as others point out, four-limb fractures are rare because it is hard to impact the ground with all four limbs [22,44]. Future studies should explain these discrepancies.

Upper limbs are another point of contention. Some articles say that arm fractures are evenly distributed among all height groups, while others find height correlation [40]. They are often associated with second-impact injuries. Fractures of the forearm can be a significant forensic clue in differentiating between pedestrian impacts and FFF, as falls are significantly more common causes of them [15]. The correlation between arm fractures and psychoactive substances was discussed in the paragraphs above.

Feet- or buttocks-first impacts are strongly linked to lower limb fractures, often bilateral. Skull fractures tend not to appear with lower limb fractures. Lower limb fractures exhibit a positive association with fall height [54]. Moreover, lower limb fractures could be important in an investigation. Articles suggest that these are much more common for suicides, most probably due to a common jump position—stepping over the edge, or run and jump [38,47,54]. In FFF, lower limb injuries are usually bilateral, which can differentiate them from pedestrian impacts [15]. Low falls have a positive correlation with proximal femur fractures [43,72]. Calcaneal ruptures are rare but may indicate high falls [18,22]. Tavone et al. hypothesize that this is due to tension in vertical deceleration, which focuses mainly on the bodies of long bones. Accordingly, in cases involving horizontal deceleration, such as motor vehicle accidents, a substantially higher frequency of foot bone fractures is observed [15].

## 8. Impact Surface and Landing Position

When the impact surface is hard and non-deformable, the victim’s body experiences sudden deceleration in an extremely short period. Thus, the energy transferred onto the skeleton is much larger; hard surface impacts cause more damage. For these reasons, hard surfaces are frequently selected by individuals attempting suicide [29,54]. Landing on a hard surface increases the chance of axial skeleton fractures and bilateral extracranial fractures. Unilateral extracranial fractures are rare in hard surface impacts. Bilateral rib and pelvis fractures positively correlate with the hard surface.

Softer impact surfaces prolong the deceleration phase and absorb greater kinetic energy, thereby reducing injury severity. Padded and soft surfaces prolong the impact, the skull vault is less likely to be damaged, and the cervical spine has a higher risk of fracture as its hyperflexion/hyperextension is prolonged [39].

The impact position decisively influences the fracture profile, with injuries clustering near the initial contact point. Clinical and biomechanical research demonstrates that feet- or buttocks-first landings are more prevalent in suicides, as evidenced by cohort studies and experimental reconstructions [73,74,75]. Conversely, head-first or whole-body impacts are more common in accidental fatal falls [22,39].

In cases of feet-first impact, injuries tend to propagate upward along the calcaneus–tibia–femur–pelvis–spine axis. Such fractures are frequently bilateral, including pilon fractures, comminuted calcaneal fractures, and lumbar burst fractures. Clinical investigations have specifically associated lumbar burst fractures with feet-first landings, thereby further supporting these injury patterns. The combined fracture profile illustrated in Figure 1 underscores a concentration in the lower extremities, pelvis, and spine; notably, the high incidence of thoracic fractures aligns with observations that chest wall injuries are present in the majority of fatal free-fall cases [1,32,47,75].

In contrast, head-first landings exert axial loads on the skull, cervical spine, and thorax. The cranium often displays highly comminuted, “spider-web” fracture patterns; the cervical and thoracic spines sustain compression injuries. As the force travels downward, pelvic injuries may also occur, as depicted in the synthesized diagram in Figure 1. Lateral or whole-body impacts result in variable fracture distributions, both unilateral and bilateral, with the thorax and spine remaining the most frequently affected regions [74,76].

In summary, the orientation of landing and the distribution of fractures are intricately associated. These observed patterns can assist in differentiating between accidental and suicidal falls, thereby serving as valuable indicators in forensic determinations of the manner of death [22,39,73].

## 9. Accident vs. Suicide

Differentiating accidents and suicides in FFF remains a difficult subject, ultimately coming down to the weighting of arguments for each case. The literature suggests many methods, often in the form of a scale [77]. Our review focused on synthesizing fracture frequencies while considering only a subset of the numerous variables. Therefore, in Table 4, we list an itemization of the above-mentioned clues.

Articles that report on homicide cases involving falls typically include only a small number of cases and often lack detailed information on injury patterns, as well as comparative data such as the victim’s sex, toxicology results, or psychiatric history. As a result, these cases were not included in our comparative analysis. Given the rarity and complexity of homicides by free fall, a dedicated multicenter study would likely be necessary to draw meaningful conclusions about this subset of cases.

The Table 4 highlights that those accidents are random events that occur in various circumstances, typically involving lower altitudes and mentally stable individuals. Due to the lower height and insufficient time to rotate in the air, the damage generally concentrates on the chest, skull, and spine, usually occurring unilaterally. Suicide victims have a psychiatric history, are often under the influence of psychoactive substances and/or alcohol, choose higher altitudes, typically land feet/buttocks-first, and develop more severe bilateral injuries.

Biomechanical analyses offer critical insight into ambiguous cases of free fatal falls. Lindgren et al. showed that computer simulations could replicate fracture patterns observed in autopsy and postmortem CT in four of five cases [78]. Furthermore, biomechanical experiments were used in the past to clarify conflicting witness accounts, distinguishing accidental from homicidal falls [14]. These findings highlight the utility of biomechanical modeling and simulation as forensic investigative tools. Some studies do not find differences between accidental and suicidal FFF. This might be due to specifics of the studied population or other intrinsic and extrinsic variables. Further studies in this area are needed [38,51].

Several authors, including Teh et al. [79] and Berghaus et al. [80], have proposed classification systems to differentiate between accidental and suicidal fatal falls from height based on fracture patterns and selected intrinsic or extrinsic variables. However, these frameworks do not account for potentially important factors such as toxicological findings, psychiatric history, the presumed landing position, or the laterality of fractures (bilateral vs. unilateral). Incorporating these variables could enhance diagnostic accuracy, particularly in cases that are borderline or ambiguous. PMCT demonstrates a markedly higher sensitivity for fracture detection compared with conventional autopsy. Whenever available, it should therefore be incorporated into the assessment of victims of fatal falls from height. PMCT is particularly valuable for identifying deep skeletal fractures that may not be apparent on autopsy alone. Such findings can provide critical insights into the direction of force transmission and, by extension, the impact orientation, thereby offering additional evidence to help clarify the circumstances and potential manner of the fall.

## 10. PMCT and Autopsy

PMCT studies on fatal falls remain relatively uncommon, and the reported fracture frequencies should be interpreted with caution. Given PMCT’s superior ability to detect skeletal injuries, studies utilizing this method are expected to report higher fracture frequencies compared to those relying on traditional autopsy. Indeed, this trend is evident in analyzed studies (see Table 5) [18,33,43,46,47]. To present the comparison in Table 5, we split the skeleton into groups: skull, chest, spine, pelvis, upper limb, and lower limb. Then, we compare the frequency of noted fractures from studies employing PMCT with those using classical autopsy reports in the established groups described in the relevant study.

PMCT’s role in forensic investigations is expanding. Fatal falls inflict the most damage to the axial skeleton, which is challenging to reveal during traditional autopsies. However, what is particularly striking is that fracture frequencies are consistently higher across all bone structures in studies using postmortem computed tomography, often by a significant margin. Few autopsy studies achieve similar numbers, but none exceed them. Autopsy-based studies may underreport certain fractures, such as pelvic fractures. Similarly, only a few studies describe pelvis or limb damage in detail. This presents a significant opportunity for future research.

## 11. Gaps in Knowledge

Precise anatomical details remain surprisingly scarce for a study focused on the distribution of fractures. While general injury locations such as the skull, upper limbs, and lower limbs are well described, data on fracture frequencies and specific bone morphologies are limited. To date, only a handful of studies have explored these aspects in detail, and even then, the findings remain incomplete.

Significant discrepancies exist among the studies included, with few conclusions free from controversy. The relationship between fall height and injury distribution across different body regions requires further investigation. In particular, skull fractures exhibit three potential distribution patterns depending on fall height: (1) bimodal, (2) linear ascending, and (3) linear descending. As reported by some works, bimodal distribution (1) has two peaks in skull fractures, below 10 and above 20 m, with a decrease in the 10–20 m range. In other papers, skull fractures rise (2) with the height of the fall or decrease (3) with it. The variables that dictate these patterns and their underlying mechanisms remain unresolved questions. A more precise classification of skull fractures—beyond the broad categories of vault, base, and facial skeleton—could provide deeper insights into fracture mechanics and potentially serve as forensic markers for determining fall height and manner of death. Similar knowledge gaps exist regarding fractures of the pelvis, spine, and limbs. For pelvic and cervical vertebral fractures, an unresolved question is whether these injuries occur more frequently in females.

Postmortem computed tomography provides an opportunity to examine these structures in greater detail, and future studies should utilize this technology to enhance fracture assessment. Another crucial area requiring additional research is the differentiation between accidental falls, suicides, and homicides. No definitive markers currently exist to distinguish these manners of death, and forensic evaluations depend on a balance of arguments for and against each scenario. Notably, the study of homicidal falls is underdeveloped due to their rarity, likely requiring a multicenter approach to gather a sufficiently large cohort for meaningful analysis.

The impact position also poses a challenge in forensic investigations, as this parameter is often difficult to determine. Differentiating between primary and secondary impact injuries could offer vital forensic insights, potentially transforming criminal investigations. Furthermore, falls onto soft surfaces are noticeably rare, underreported, and often examined only in isolated case studies. A more detailed understanding of how various impact surfaces influence injury patterns could further refine forensic interpretations. Similarly, forensic experts encounter challenges in assessing the distance between the deceased and the high-altitude structure from which the victim has fallen, as this information can provide critical insights into the victim’s intent or motivations. The interpretation of this distance is complicated by distinguishing between injuries sustained from the primary impact (the initial ground contact) and those that arise from secondary impacts (for instance, collisions with intermediate surfaces). Without a comprehensive understanding of the injury patterns associated with primary versus secondary impacts, this potentially significant clue may be misinterpreted.

An important yet underappreciated gap lies in the limited clinical integration of postmortem fracture pattern data—particularly from PMCT—into trauma diagnostics and treatment algorithms. Despite the clear potential of such data to inform clinical decision-making, especially in unconscious or polytraumatized patients with unclear injury mechanisms, no current guidelines systematically incorporate forensic imaging insights. Future interdisciplinary studies are needed to translate anatomical and forensic findings into actionable clinical protocols, thereby improving trauma care and patient outcomes.

## 12. Limitations

This review has several limitations. First, despite a comprehensive and systematic search strategy, relevant studies may have been inadvertently missed, particularly those published in non-English languages, which were excluded from the analysis. This language restriction may have introduced selection bias and limited the geographical diversity of the findings.

Second, the reviewed literature demonstrates considerable heterogeneity in terms of sample sizes, fall height categorization, injury reporting methods, and classification of the manner of death. These inconsistencies hindered quantitative meta-analysis and necessitated descriptive synthesis. Moreover, many studies lacked standardized definitions for skeletal regions or combined multiple injury types into single categories (e.g., “chest injuries”), complicating cross-study comparisons.

Third, PMCT—despite its recognized diagnostic advantages—was underrepresented in the included studies, accounting for only 14.3% of the analyzed papers. As a result, conclusions regarding the comparative effectiveness of PMCT and autopsy should be interpreted cautiously and require further validation through prospective, standardized studies. Moreover, the included studies did not follow a unified protocol for PMCT assessment of FFF. Similarly, the computed tomography scanners used varied between studies. As a result, the reported fracture types and patterns may differ slightly, introducing potential bias.

Additionally, important contextual variables such as body mass index (BMI), victim occupation, or precise impact position were rarely reported, limiting the ability to assess their influence on fracture patterns. Toxicological data were inconsistently presented, with many studies lacking detailed information on alcohol or drug use, and few differentiating between accidental and suicidal cases in this regard. Comorbidities and pre-existing conditions (such as osteoporosis) were not universally reported. Thus, their influence over the FFF fracture pattern could not be studied. Finally, the exclusion of grey literature and studies with fewer than 10 cases, though methodologically justified, may have omitted valuable case-level insights, particularly for rare injury types or unique forensic scenarios. During this study, we also encountered cases that did not lend themselves to straightforward classification, such as falls from height without resulting in lethal injuries or fatalities, or those caused by secondary mechanisms, including impalement on objects during descent. These cases were excluded from the present analysis; however, their occurrence highlights the heterogeneity of fall-related fatalities and the difficulty of capturing all possible scenarios within a rigid analytical framework. This underlines the need for cautious interpretation in forensic practice, where atypical presentations may fall outside established classificatory schemes yet still provide valuable contextual information [81,82].

## 13. Key Takeaways

- Fractures in FFF are dispersed throughout the entire skeleton, with a predominance in the axial skeleton.
- Males > females both in accidental and suicidal FFF.
- Variables associated with suicide: intoxication, psychiatric history.
- No definitive connection between fracture pattern and intoxication in FFF, possible correlation of cause of death and upper limb injury.
- Factors influencing fracture pattern are the height of the fall, impact surface, cause of the fall, sex.
- General fracture frequencies: chest > skull > limbs > pelvis > spine.
- There are no specific indicators that the death is accidental or suicidal, only arguments for and against those hypothesizes.
- Accident damage profile: chest > skull > spine > pelvis > limbs.
- Suicide damage profile: chest > pelvis/skull > limbs.

## 14. Conclusions

This review synthesizes skeletal injury patterns in FFF, integrating autopsy and PMCT findings. Chest, skull, and pelvic fractures were the most common, with suicidal falls resulting in more pelvic and skull injuries, and accidental falls causing more thoracic trauma. Both intrinsic factors (e.g., sex, psychiatric disorders, intoxication) and extrinsic factors (e.g., fall height, surface type, body position) influenced the distribution of fractures.

PMCT demonstrated higher sensitivity than autopsy, particularly for complex structures such as the spine and pelvis, supporting its integration into standard forensic workflows. Methodological inconsistencies in reporting fall height, injury types, and cause of death remain; future studies should standardize protocols and investigate underexplored factors.

Knowledge of typical fracture patterns and PMCT data can aid in differential diagnosis and distinguish fall-related injuries from other trauma mechanisms.

## Figures and Tables

**Table 1:** Intrinsic variables (victim-related: sex, manner of death, toxicology, and psychiatric history) influencing FFF injuries, collected from the articles and presented in chronological order. U = unknown.

Authors, Year,	Reported FFF with Postmortem Examination [N]	Male Victims	Female Victims	Suicidal Deaths	Male Suicidal Deaths	Female Suicidal Deaths	Accidental Deaths	Accidental Deaths Among Males	Accidental Deaths Among Females	Homicides	Unknown Cause of the Fall		Alcohol Detected		Prescribed/Resuscitation Medications		Overdose/Substance Abuse Disorder	Depresive Disorder	Schizophrenia	Other
Goonetilleke et al., 1980 [22]	146	73.97% (108)	26.03% (38)	25.2% (37)	51.35% (19)	48.65% (19)	56.85% (83)	89.16% (74)	10.84% (9)	0%	17.81% (26)	U	U	U	U	10.27%	U	U	U	U
Lucas et al., 1981 [23]	100	56% (56)	44% (44)	100% (100)	56% (56)	44% (44)	0%	0%	0%	0%	0%	U	U	U	U	U	U	U	U	U
Gupta et al., 1982 [24]	63	65.8% (41)	28.57% (18)	42.86% (27)	55.56% (15)	44.44% (12)	49.21% (31)	83.87% (26)	16.13% (5)	1.59% (1)	6.35% (4)	U	U	U	U	U	U	U	U	U
Simonsen et al., 1983 [25]	10	70% (7)	30% (3)	100% (10)	70% (7)	30% (3)	0%	0%	0%	0%	0%	U	U	U	U	U	U	U	U	U
Hanzlick et al., 1990 [26]	14	78.94% (15)	21.05% (4)	100% (14)	78.95% (15)	21.05% (4)	0%	0%	0%	0%	0%	10.53% (2)	10.53% (2)	U	U	U	U	U	U	U
MAJ Mark Lafave et al., 1995 [27]	281	85% (239)	15% (42)	100% (281)	85% (239)	15% (42)	0%	U	U	0%	0%	U	U	U	U	U	U	U	U	U
Cetin et al., 2001 [28]	20	93.8% (61)	6.2% (4)	100% (20)	93.8% (61)	6.2% (4)	0%	0%	0%	0%	0%	U	U	U	U	20% (4)	10% (2)	U	U	10% (2)
Goren, Subasi et al., 2003 [29]	484	61.2% (296)	38.8% (188)	11.0% (53)	45.3% (24)	54.7% (29)	89.0% (431)	63.1% (272)	36.9% (159)	0%	0%	U	U	U	U	4.55% (22)	0.78% (3)	2.27% (11)	0.83% (4)	0.78% (3)
Tu¨rk et al., 2004 [30]	68	72.06% (49)	27.94% (19)	50% (34)	56% (19)	44% (15)	34% (23)	91% (21)	9%	0%	16% (11)	33.92% (23)	14.71% (10)	13.24% (9)	6% (4)	32.35% (22)	8.82% (6)	7.35% (5)	1.47% (1)	10.29% (10)
Kohli, Banerjee, 2006 [31]	151	88.74% (134)	11.26% (17)	U	U	U	U	U	U	U	U	U	U	U	U	U	U	U	U	U
Venkatesh et al., 2007 [32]	80	85% (68)	15% (12)	3.75% (3)	U	U	95% (76)	U	U	1.25% (1)	U	10% (8)	10% (8)	U	U	2.5% (2)	U	U	U	U
Weilemann et al., 2007 [33]	20	55% (11)	45% (9)	80% (16)	50% (8)	50% (8)	15% (3)	66.33%	33.33%	0%	5% (1)	U	U	U	U	U	U	U	U	U
Atanasijevic et al., 2009 [34]	660	469 71% (469)	29% (191)	56% (370)	U	U	44% (290)	U	U	0%	0%	U	U	U	U	U	U	U	U	U
Behera et al., 2010 [35]	174	60.9% (106)	39.1% (68)	0%	0%	0%	100% (174)	60.9% (106)	39.1% (68)	0%	0%	U	U	U	U	U	U	U	U	U
Steffen et al., 2010 [18]	10	90% (9)	10% (10)	0%	0%	0%	100% (10)	90% (9)	10% (1)	0%	0%	U	U	U	U	U	U	U	U	U
Thierauf et al., 2010 [36]	123	81.3% (100)	18.7% (23)	27.64% (34)	64.71% (22)	35.29% (12)	72.36% (89)	87.64% (78)	12.36% (11)	0%	0%	24.39% (30)	24.39% (30)	U	U	U	U	U	U	U
Papadopoulos et al., 2011 [37]	970	64.54% (626)	35.46% (344)	26.5% (257)	U	U	60.41% (586)	U	U	0.93% (9)	12.17% (118)	12.58% (122)	U	U	U	U	U	U	U	U
Petaros et al., 2013 [38]	179	U	U	54% (96)	U	U	46% (83)	U	U	0%	0%	U	U	U	U	U	U	U	U	U
Freeman et al., 2013 [39]	1002	74.3% (1210)	25.7% (418)	61.4% (270)	U	U	28% (1234)	U	U	4.5% (6)	U	U	U	U	U	U	U	U	U	U
Casali et al., 2014 [40]	307	61% (188)	39% (119)	U	U	U	U	U	U	U	U	U	U	U	U	U	U	U	U	U
Obeid et al., 2016 [41]	423	76.6% (324)	23.4% (99)	71% (291)	U	U	29% (119)	U	U	<1% (1)	0%	U	U	U	U	U	U	U	U	U
Rao et al., 2016 [42]	73	80.8% (59)	19.2% (14)	5.5% (4)	U	U	94.5% (69)	U	U	0%	0%%	U	U	U	U	U	U	U	U	U
Rowbotham et al., 2017 [43]	145	59% (86)	41% (59)	3% (4)	U	U	97% (141)	U	U	0%	0%	U	U	U	U	U	U	U	U	U
Abder-Rahman et al., 2017 [44]	352	72.7% (256)	27.3% (96)	8.8% (31)	U	U	86.1% (303)	U	U	0.6% (2)	4.5% (16)	U	U	U	U	U	U	U	U	U
Eş et al., 2017 [45]	170	70.6% (120)	29.4% (50)	30.6% (52)	U	U	69.4% (118)	U	U	0%	0%	81.18% (138)	5.29% (9)	2.35% (4)	73.52% (125)	U	U	U	U	U
Heimer et al., 2018 [46]	44	72.7% (32)	27.3% (12)	47.7% (21)	U	U	22.7% (10)	U	U	0%	29.5% (13)	U	U	U	U	U	U	U	U	U
Rowbotham et al., 2018 [47]	95	76.8% (73)	23.2% (22)	78.9% (75)	U	U	20% (19)	U	U	1.1% (1)	0%	64.21%	U	U	U	U	U	U	U	U
Türkoğlu et al., 2019 [48]	213	67.6% (144)	32.4% (69)	19.2% (41)	51.22% (21)	48.78% (20)	80.3% (171)	71.35% (122)	28.66% (49)	0.5% (1)	0%	1.41% (3)	0.94% (2)	0.47% (1)	U	15.02% (32)	U	6.1% (13)	U	8.92% (19)
Casali et al., 2019 [49]	385	54.55% (210)	45.45% (175)	U	U	U	U	U	U	U	U	U	U	U	U	U	U	U	U	U
Çakı et al.,2020 [50]	206	82.5% (170)	17.5% (36)	11.2% (23)	65.22% (15)	34.78% (8)	86.4% (178)	85.96% (153)	14.05% (25)	2.4% (5)	0%	U	U	U	U	U	U	U	U	U
Ramadan et al., 2020 [51]	42	76.2% (32)	23.8% (10)	9.50% (4)	U	U	66.7% (28)	U	U	4.8% (2)	9.52% (4)	2.4%	0%	0%	2.4%	9.52% (4)	U	U	U	U
Tsellou et al., 2022 [52]	261	73.9% (193)	26.1% (68)	53.6% (140)	68.4% (96)	31.43% (44)	37.5% (98)	U	U	0%	8.9% (23)	U	U	U	U	55.94% (146)	2.68% (7)	24.14% (63)	U	31.03% (81)
Kandeel et al., 2022 [53]	53	76.3% (39)	26.4% (14)	1.9% (1)	U	U	98.1% (51)	U	U	0%	0%	U	U	U	U	U	U	U	U	U
Chelly et al., 2023 [54]	141	85.8% (121)	14.2% (20)	13.48% (19)	68.4% (13)	31.6% (6)	86.52% (122)	73.05% (103)	7.8% (11)	0%	0%	3.55% (5)	2.13% (3)	U	1.42% (2)	10.64% (15)	U	U	U	U
Tavone et al., 2024 [15]	129	U	U	U	U	U	U	U	U	U	U	U	U	U	U	U	U	U	U	U

**Table 2:** Extrinsic variables (event-related: fall height, type of landing surface) influencing FFF injuries and use of postmortem imaging, collected from the articles and presented in chronological order. U = unknown; *—Classified in the source article as ‘other’ or as cases not easily categorized (e.g., wells with an indeterminate water level).

Author, Year	Analyzed Heights (m)	Dominating Height Group	Nondeformable Surfaces	Deformable Surfaces	Water	Other *	PMCT Use
Goonetilleke et al., 1980 [22]	0.76–51.82 m	3.048–4.572 m (26.03%)	U	U	U	U	no
Lucas et al., 1981 [23]	76.2–79.55 m	76.2–79.55 m (100%)	0%	0%	100%	0%	no
Gupta et al., 1982 [24]	0–27.43 m	3.3528–6.096 m (25.43%)	100%	0%	0%	0%	no
Simonsen et al., 1983 [25]	35–51 m	30–40 m (90%)	0%	0%	100%	0%	no
Hanzlick et al., 1990 [26]	18–141 m	60–75 m (50%) *	100%	0%	0%	0%	no
MAJ Mark Lafave et al., 1995 [27]	76.2–79.55 m	76.2–79.55 m (100%)	0%	0%	100%	0%	no
Cetin et al., 2001 [28]	64 m	65 m (100%)	0%	0%	100%	0%	no
Goren, Subasi et al., 2003 [29]	1–28 m	1–5 m (50.41%)	U	U	U	U	no
Tu¨rk et al., 2004 [30]	3–57 m	6–10 m (20.59%)	U	U	U	U	no
Kohli, Banerjee, 2006 [31]	3–<15 m	3–6 m (62.9%)	U	U	U	U	no
Venkatesh et al., 2007 [32]	0.6–23 m	0–3.048 m (37.5%)	U	U	U	U	no
Weilemann et al., 2007 [33]	5–70 m	30–40 m (45%)	100%	0%	0%	0%	yes
Atanasijevic et al., 2009 [34]	0–70 m	<7 m (45.61%)	100%	0%	0%	0%	no
Behera et al., 2010 [35]	0.6–12 m	5.38 m	U	U	U	U	no
Steffen et al., 2010 [18]	U	U	100%	0%	0%	0%	yes
Thierauf et al., 2010 [36]	1.5–100 m	U	U	U	U	U	no
Papadopoulos et al., 2011 [37]	1–200 m	U	U	U	U	U	no
Petaros et al., 2013 [38]	1.5–101 m	4–10 m (35.75%)	100%	0%	0%		no
Freeman et al., 2013 [39]	<3 m. >3 m	U	U	U	U	U	no
Casali et al., 2014 [40]	3–84 m	<12 m (51.8%)	solid				no
Obeid et al., 2016 [41]	1.83–128.02 m	15.24 m–30.1752 m (39.72%)	U	U	U	U	no
Rao et al., 2016 [42]	U	U	U	U	U	U	no
Rowbotham et al., 2017 [43]	U	U	99%	1%	0%	0%	yes
Abder-Rahman et al., 2017 [44]	1.5–15 m	>9 m (38.6%)	U	U	U	U	no
Eş et al., 2017 [45]	3–60 m	U	U	U	U	U	no
Heimer et al., 2018 [46]	3.3 m-70 m	3–10 m (36.36%) = 20–70 m (36.36%)	100%	0%	0%	0%	yes
Rowbotham et al., 2018 [47]	>3–>51 m	3–25 m (51.6%)	86.3%	13.7%	0%	0%	yes
Türkoğlu et al., 2019 [48]	<5 m >5 m	<5 m (52.6%)	U	U	U	U	no
Casali et al., 2019 [49]	<6 m–36 m	equinumerous groups	100%	0%	0%		no
Çakı et al., 2020 [50]	8.2 m ± 0.7 m	U	U	U	U	U	no
Ramadan et al., 2020 [51]	<6.01 m >6.01 m	>6.01 m (78.6%)	U	U	U	U	no
Tsellou et al., 2022 [52]	9.35 ± 7.35 m	U	U	U	U	U	no
Kandeel et al., 2022 [53]	<6 m >6 m	>6 m (56.6%)	U	U	U	U	no
Chelly et al., 2023 [54]	3–>15 m	3–6 m (41.8)	77.30%	0%	0.00%	22.7%	no
Tavone et al., 2024 [15]	U	U	U	U	U	U	no

**Table 3:** Risk of bias assessment using NOS.

Study	Design	N	PMCT_Used	NOS_Selection_(0–4)	NOS_Comparability_(0–2)	NOS_Outcome_(0–3)	NOS_Total_(0–9)	Overall_Risk_of_Bias
Goonetilleke et al. (1980) [22]	Retrospective autopsy series	146	No	2	2	2	6	Moderate
Lucas et al. (1981) [23]	Retrospective autopsy series	100	No	2	2	2	6	Moderate
Gupta et al. (1982) [24]	Retrospective autopsy series	63	No	2	2	1	5	Moderate
Simonsen et al. (1983) [25]	Retrospective autopsy series	10	No	2	2	1	5	Moderate
Hanzlick et al. (1990) [26]	Retrospective autopsy series	14	No	2	2	1	5	Moderate
Lafave et al. (1995) [27]	Retrospective autopsy series	281	No	3	2	2	7	Low
Cetin et al. (2001) [28]	Retrospective autopsy series	20	No	2	2	1	5	Moderate
Goren et al. (2003) [29]	Retrospective autopsy series	484	No	3	2	2	7	Low
Türk & Tsokos (2004) [30]	Retrospective autopsy series	68	No	2	2	1	5	Moderate
Kohli & Banerjee (2006) [31]	Retrospective autopsy series	151	No	2	2	2	6	Moderate
Venkatesh et al. (2007) [32]	Retrospective autopsy series	80	No	2	2	1	5	Moderate
Weilemann et al. (2007) [33]	Retrospective autopsy series with PMCT	20	Yes	2	2	1	5	Moderate
Atanasijevic et al. (2009) [34]	Retrospective autopsy series	660	No	3	2	2	7	Low
Behera et al. (2010) [35]	Retrospective autopsy series	174	No	2	2	2	6	Moderate
Steffen et al. (2010) [18]	Retrospective autopsy series with PMCT	10	Yes	2	2	1	5	Moderate
Thierauf et al. (2010) [36]	Retrospective autopsy series	123	No	2	2	2	6	Moderate
Papadopoulos et al. (2011) [37]	Retrospective autopsy series	970	No	3	2	2	7	Low
Petaros et al. (2013) [38]	Retrospective autopsy series	179	No	2	2	2	6	Moderate
Freeman et al. (2013) [39]	Retrospective autopsy series	1002	No	3	2	2	7	Low
Casali/Bruno et al. (2014) [40]	Retrospective autopsy series	307	No	3	2	2	7	Low
Obeid et al. (2016) [41]	Retrospective autopsy series	423	No	3	2	2	7	Low
Rao et al. (2016) [42]	Retrospective autopsy series	73	No	2	2	1	5	Moderate
Rowbotham et al. (2017) [43]	Retrospective autopsy series with PMCT	145	Yes	2	2	2	6	Moderate
Abder-Rahman et al. (2017) [44]	Retrospective autopsy series	352	No	3	2	2	7	Low
Eş et al. (2017) [45]	Retrospective autopsy series	170	No	2	2	2	6	Moderate
Heimer et al. (2018) [46]	Retrospective autopsy series with PMCT	44	Yes	2	2	1	5	Moderate
Rowbotham et al. (2018) [47]	Retrospective autopsy series with PMCT	95	Yes	2	2	1	5	Moderate
Türkoğlu et al. (2019) [48]	Retrospective autopsy series	213	No	3	2	2	7	Low
Casali et al. (2019) [49]	Retrospective autopsy series	385	No	3	2	2	7	Low
Çakı et al. (2020) [50]	Retrospective autopsy series	206	No	3	2	2	7	Low
Ramadan et al. (2020) [51]	Retrospective autopsy series	42	No	2	2	1	5	Moderate
Tsellou et al. (2022) [52]	Retrospective autopsy series	261	No	3	2	2	7	Low
Kandeel & Azab (2022) [53]	Retrospective autopsy series	53	No	2	2	1	5	Moderate
Chelly et al. (2023) [54]	Retrospective autopsy series	141	No	2	2	2	6	Moderate
Tavone et al. (2024) [18]	Retrospective autopsy series	129	No	2	2	2	6	Moderate

**Table 4:** A comparison of the characteristics of FFF as a result of accident and suicide—note that when we write “Bilateral upper limb fractures,” we mean that it is statistically significantly more common in one group, not completely unseen. “+” indicates a positive correlation; “-” indicates a negative correlation.

Argument	Accident	Suicide	Citation
Intoxication	-	+	[30,45,47,57]
Psychiatric history	-	+	[22,29,41,52]
High altitude	-	+	[29,32,37,47,52]
More severe, multifocal injuries	-	+	[38,47]
Feet/buttocks first impact	-	+	[47,54]
Side/whole body/headfirst impact	+	-	[33]
Fracture pattern: Chest > Skull > Spine > pelvis > limbs	+	-	[18,23,25,27,38,41,50,52]
Fracture pattern: Chest > pelvis/skull > limbs	-	+	[18,23,25,27,38,41,50,52]
Pelvis fractured	-	+	[37,38]
Bilateral lower limb fractures	-	+	[47,54]
Bilateral upper limb fractures	-	+	[38,47]
Severe bilateral chest fractures	-	+	[38,47]

**Table 5:** Comparison between PMCT and autopsy-detected studies.

Skeleton Part	PMCT	Autopsy
Skull	83%	53%
Chest	95%	62%
Spine	79%	19%
Pelvis	83%	24%
Upper limbs	78%	21%
Lower limbs	69%	28%

## Data Availability

No new data were created or analyzed in this study.

## Abbreviations

The following abbreviations are used in this manuscript:

FFF	fatal free fall
BFT	blunt force trauma
WHO	World Health Organization
MVA	motor vehicle accidents
BMI	body mass index
PMCT	postmortem computed tomography
PE	potential energy
AI	artificial intelligence
NOS	Newcastle–Ottawa Scale
